# The network organization of protein interactions in the spliceosome is reproduced by the simple rules of food-web models

**DOI:** 10.1038/srep14865

**Published:** 2015-10-07

**Authors:** Mathias M. Pires, Maurício Cantor, Paulo R. Guimarães, Marcus A. M. de Aguiar, Sérgio F. dos Reis, Patricia P. Coltri

**Affiliations:** 1 Departamento de Ecologia, Instituto de Biociências, 05508-090, Universidade de São Paulo, São Paulo, Brazil; 2 Department of Biology, Dalhousie University, Halifax, Nova Scotia B3H 4J1, Canada; 3 Departamento de Física da Matéria Condensada, Instituto de Física ‘Gleb Wataghin’, 13083-859, Universidade Estadual de Campinas, Campinas, Brazil; 4 Departamento de Biologia Animal, Instituto de Biologia, 13083-970, Universidade Estadual de Campinas, Campinas, Brazil; 5 Departamento de Biologia Celular e do Desenvolvimento, Instituto de Ciências Biomédicas, 05508-000, Universidade de São Paulo, São Paulo, Brazil

## Abstract

The network structure of biological systems provides information on the underlying processes shaping their organization and dynamics. Here we examined the structure of the network depicting protein interactions within the spliceosome, the macromolecular complex responsible for splicing in eukaryotic cells. We show the interactions of less connected spliceosome proteins are nested subsets of the connections of the highly connected proteins. At the same time, the network has a modular structure with groups of proteins sharing similar interaction patterns. We then investigated the role of affinity and specificity in shaping the spliceosome network by adapting a probabilistic model originally designed to reproduce food webs. This food-web model was as successful in reproducing the structure of protein interactions as it is in reproducing interactions among species. The good performance of the model suggests affinity and specificity, partially determined by protein size and the timing of association to the complex, may be determining network structure. Moreover, because network models allow building ensembles of realistic networks while encompassing uncertainty they can be useful to examine the dynamics and vulnerability of intracelullar processes. Unraveling the mechanisms organizing the spliceosome interactions is important to characterize the role of individual proteins on splicing catalysis and regulation.

Networks are pervasive across all levels of biological organization. Protein-protein networks describe interactions among proteins in a given cellular compartment or macromolecular complex^1^,^2^, metabolic networks describe biochemical pathways within cells^3^, social networks delineate the structure of human and animal societies^4^ and ecological networks such as food webs are networks that depict trophic interactions among species^5^. The ways in which each element (proteins, individuals, and species) within those systems connects to each other give rise to organizational patterns, which in turn affect the system functioning^6^,^7^.

Here we investigate the structural properties of a sub-cellular network describing interactions between proteins of the spliceosome in *Saccharomyces cerevisiae*^8^. The spliceosome is the macromolecular machinery responsible for splicing in eukaryotic cells. This dynamic complex is composed of small nuclear RNAs (snRNAs) and more than 100 proteins, and its catalytic activity is dependent upon dynamic interactions among its components^9^. Splicing is an important step on gene regulation and, consistently, splicing defects have been associated with development of diseases including different types of cancer^10^. Important information on the regulation and vulnerability of splicing may be encoded in the structure of the spliceosome networks. Therefore, we first explore two network properties that both theoretical and empirical studies suggest may affect the dynamics of distinct biological networks: modularity and nestedness.

In modular networks elements are organized in semi-independent groups. Modularity is observed in disparate biological systems, including metabolic pathways^11^, animal societies^12^, and food webs^5^. Conversely, a network has a nested strucuture when the set of elements interacting with poorly-connected elements also interact with highly-connected elements. Nestedness has also been detected in several biological systems such as animal-plant mutualistic networks^13^, predator-prey interaction networks^14^, networks describing how populations are distributed across sites^15^, and patterns of diet overlap among individuals within populations^16^. Although nestedness and modularity represent radically distinct network patterns, variable levels of nestedness and modularity can be observed in a single network^17^. We used spectral analysis^18^ to investigate different possible configurations of the network, incorporating the inherent uncertainty of the characterization of protein-protein networks, and explored the structure of the spliceosome network.

We then investigated the proximate causes leading to the emergence of modularity and nestedness in the spliceosome network. Protein-protein interactions often depend on the interplay between the affinity and specificity of receptors and particular areas of interacting proteins^19^. Affinity and specificity are very general ingredients and also shape patterns of interaction in other biological networks. For example, the assembly of ecological networks depends, essentially, on trait complementarity (affinity) between resources and consumers and specificity in consumers’ diets. The study of food webs has benefited from assembly models that are able to generate theoretical networks structurally similar to empirical food webs^20^ by using simple sets of rules based on trait complementarity and specificity^20^,^21^,^22^.

Thus, to gain further insights on the assembly mechanisms organizing spliceosome protein interactions, we adapted the probabilistic niche model^23^ (PNM), and tested whether the rules shown to reproduce ecological networks are able to reproduce the structural properties of the spliceosome network. If the underlying mechanisms shaping the assembly of the spliceosome network can be defined in terms of complementarity and specificity, we would expect the performance of the model in reproducing the spliceosome network would be comparable to its performance in reproducing food webs. We then used the estimated parameters of the best-fitted models to gain insights on the organization of the spliceosome network. We cross-validated the patterns we detected by performing similar analysis using other two datasets describing interactions between core spliceosomal proteins in *S. cerevisiae* and humans.

## Results

**Figure 1:**
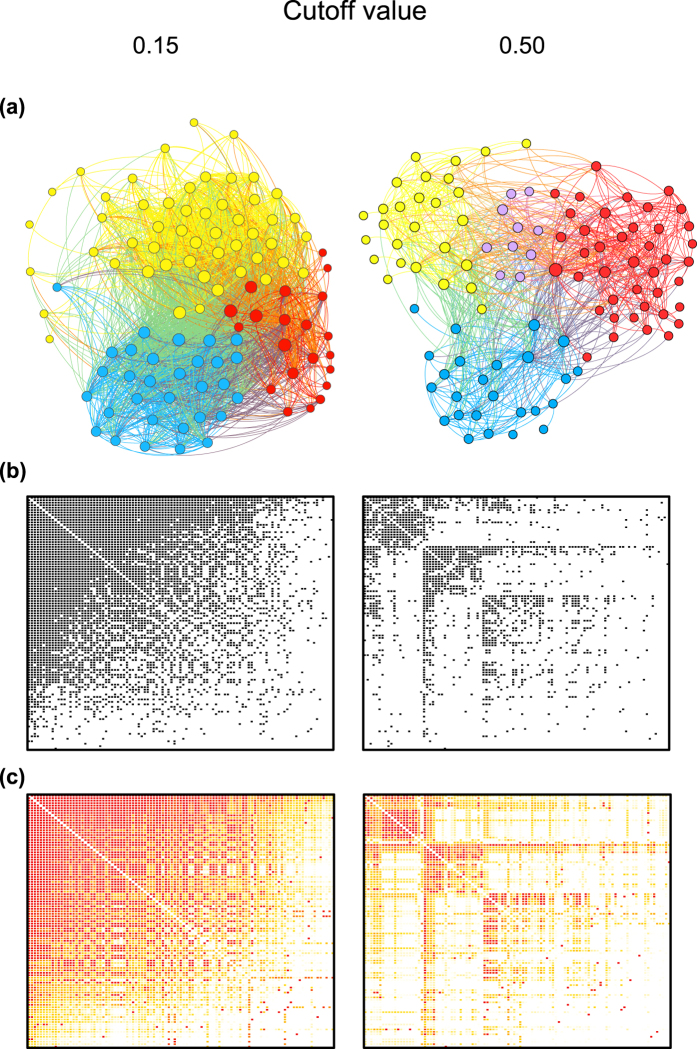
The yeast spliceosome networks. (**a**) Spliceosome protein-protein networks with two different cutoffs defining interactions (0.15 and 0.5). Nodes represent proteins and links represent the interactions among them. Node size is proportional to the protein connectivity (number of interactions a protein establishes with others). Different colors represent different network modules. Networks were built using Gephi (http://gephi.org). (**b**) Matrix representation of the empirical protein-protein interactions for each cutoff. Each row or column represents a protein, and the black squares represent an interaction between two proteins. (**c**) Matrix representation of protein-protein interactions yielded by the model. The color heat of squares corresponds to the probability of interactions according to the model. Note the correspondence between the observed interactions (**b**) and interactions predicted by the model (**c**).

The *S. cerevisiae* spliceosome network analyzed contained 103 proteins. Spectral network analysis (see methods) showed that the description of structure was affected by the level of interaction reliability considered to build the network. With a permissive filtering of the network, in which we kept all the interactions with reliability greater than 0.15 (cutoff = 0.15; see Methods) the resulting network contained 2,538 interactions out of the 5,253 possible interactions (network connectance, *C* = 0.48; Fig. 1). The average connectivity of proteins was 49.28 ± 24.08 interactions per protein, i.e. a highly cohesive network. When we were more restrictive in filtering the network and considered only interactions with reliability greater than 0.5 (cutoff = 0.5), the number of interactions was reduced to 881 (*C* = 0.17), resulting in a sparser network with 17.10 ± 13.04 interactions per protein. In both configurations analyzed, larger proteins (inferred by their estimated molecular weight, MW) were more likely to have more interactions (0.15 cutoff: slope = 0.19, *p* < 0.001; 0.5 cutoff: slope = 0.09; *p* < 0.01; Supplementary Fig. S2). Moreover, the number of interactions of each protein in the networks built under the two different filtering schemes was correlated, suggesting the differences in connectivity among proteins were preserved between cutoffs (*r* = 0.62, *p* < 0.001; Supplementary Fig. S3).

The spliceosome network was significantly nested regardless of the cutoff value used to build the network (*N*_*0.15*_ = 0.75, *p* < 0.01; *N*_*0.5*_ = 0.38, *p* < 0.01). Interactions between spliceosome proteins also generate a modular structure (*M*_*0.15*_ = 0.12, *p* < 0.01; *M*_*0.5*_ = 0.32, *p* < 0.01) with proteins divided among three modules for cutoff = 0.15, and four modules for cutoff = 0.5. The degree of nestedness decreased and modularity increased as we increased the cutoff values for interaction reliability (Supplementary Fig. S4). We then analyzed interactions among core components of both yeast and human spliceosomes sampled from distinct databases to investigate if our results are dependent of data source, species identity and the definition of component proteins. The structural patterns of these subnetworks were more similar to the main network using cutoff = 0.5. Both the *S*. *cerevisiae* and human core networks were smaller than those considered in the baseline analyses, much sparser (*C* < 0.1) and highly modular (*M*_*y*_ = 0.59; *M*_*h*_ = 0.71).

**Figure 2:**
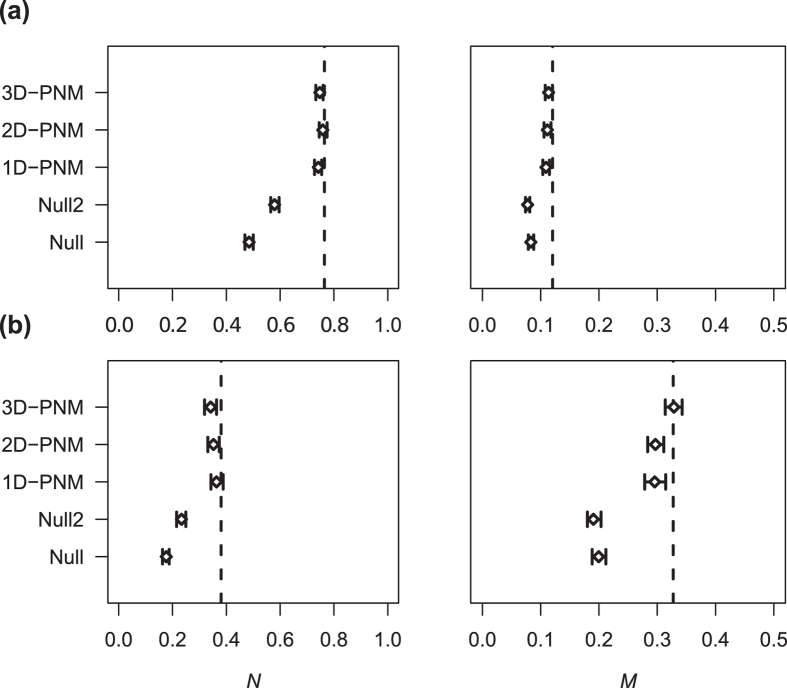
Structure of empirical and model networks. Nestedness (*N*) and modularity (*M*) of theoretical networks built according to two null models and three versions of the probabilistic niche model, PNM. Whiskers = 95% CI. The dashed lines represent estimates for the observed networks. (**a**) Results for all interactions with reliability >0.15. (**b**) Results considering only interactions with reliability >0.5.

We tested the performance of food-web models with varying levels of complexity (one-, two- and three-dimensional niche models, and two null models) in reproducing the interactions and overall structure of the spliceosome network. The three versions of the probabilistic niche model (1D-, 2D- and 3D-PNM) were able to correctly predict more than 80% of the protein-protein interactions in the main spliceosome network (Fig. 1). This result was consistent regardless the cutoff considered to build the network. When considering only the core proteins compiled from two different databases, the performance of the PNM was even better, with an expected number of correctly predicted interactions close to 99% (Supplementary Table S1). Increasing the dimensionality of the PNM increased goodness of fit for the main *S. cerevisiae* network (Table 1), but the 1D-PNM had a superior performance for the subset of core *S. cerevisiae* spliceosomal proteins and the core of human spliceosome proteins (Supplementary Table S1). Altogether all three PNM versions performed better in reproducing the interaction patterns of individual proteins than simpler (null) models that assumed the probabilities of pairwise interactions were equiprobable (null) or proportional to the number of interactions of potentially interacting proteins (null 2; Table 1). Moreover, all versions of the PNM performed well in reproducing the structure of the observed network, whereas networks generated by the two simpler models did not reproduce nestedness and modularity patterns (Fig. 2 and Supplementary Fig. S5).

**Figure 3:**
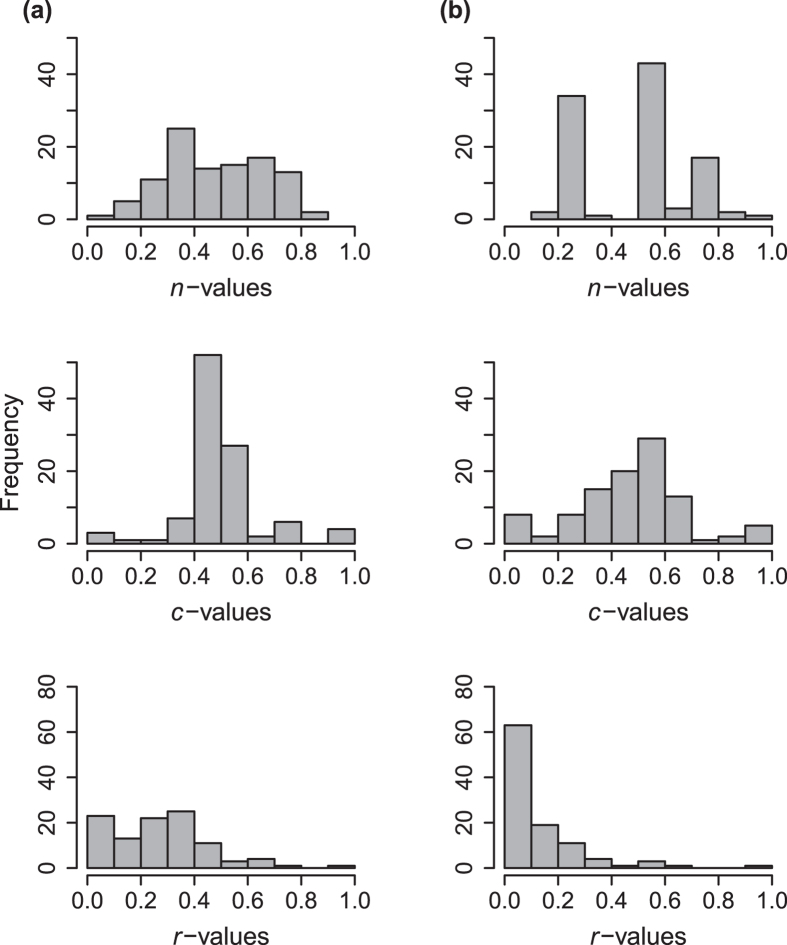
Distributions of model parameters for the one-dimensional probabilistic niche model (1D-PNM). *n*-values represent the position of proteins in a trait axis; *c*-values represent the center of the interaction range of each protein along the trait axis; *r*-values represent the interaction range of a given protein. Values correspond to the maximum likelihood parameter set. (**a**) Results for all interactions with reliability >0.15. (**b**) Results considering only interactions with reliability >0.5.

The performance of the one-dimensional PNM in reproducing network patterns was almost as good as that of more complex models including more trait axes (Table 1). To understand how the model was able to reproduce network structure, we evaluated the distribution of the best-fitted parameters for the one-dimensional PNM. Model parameters determine the position of each protein *i* in the trait axis, *n*_*i*_, the center of its interaction range, *c*_*i*_, and the length of its interaction range, *r*_*i*_. Although we started from uniformly distributed values to find the maximum likelihood estimates, the distributions of these parameters differed after optimization. For cutoff = 0.15, the positions of proteins in the trait axis, *n*-values, were distributed almost uniformly between 0.1 and 0.85. Yet, for cutoff = 0.5 we see three peaks of *n*-values (Fig. 3). *c*-values, on the other hand, followed a bell-shaped distribution for both cutoff values, although variance was greater for cutoff = 0.5. Lastly, the distribution of *r*-values was skewed, with a slow decay for cutoff = 0.15, but a fast decay for cutoff = 0.5 (Fig. 3).

## Discussion

Spliceosome proteins of *S. cerevisiae* form an interaction network that is simultaneously nested and modular, although the nestedness-modularity balance may depend on the interaction reliability considered. Similar patterns are observed in ecological networks describing species interactions^17^,^24^ or how individuals of distinct populations share resources^25^. Although for the core spliceosomal proteins we found no significant nestedness, when considering the whole network our results suggest that the interactions of proteins that establish few connections are nested subsets of the interactions of highly connected proteins, as previously described for interactions among proteins participating in other intracellular processes^26^. It is well known that proteins vary widely in their specificity; however, the degree of nestedness is higher than expected by the simple variation in the degree of specificity.

The observation that spliceosome networks show significant level of nestedness leads to two predictions on the robustness of spliceosome. First, nested networks are expected to be very robust to random loss of nodes, but very dependent on the core of highly connected nodes^27^. Therefore, we hypothesize that nestedness provides an additional layer of robustness to the spliceosome organization, but also imply that mutations that affect the interactions of core proteins will have unanticipated large effects – larger than expected by their number of interactions – on the splicing process. Performing series of mutation and knock-down experiments and then correlating the changes of splicing efficiency with the number of interactions of the protein analyzed could be a test for this prediction. If nestedness matters for splicing robustness we should expect that the effects of knocking down a highly connected protein would lead to a severe impairment in splicing efficiency, larger than expected solely by its number of interactions. In fact, loss of core components such as Prp8p for example, can have dramatic implications for spliceosome assembly, being associated to development of pathologies such as *retinitis pigmentosa*^28^. Second, nestedness at the network level also implies multiple asymmetries in which some poorly connected proteins are strongly associated to the core of proteins, making these networks slightly dissortative^29^. We expect that knocking down poorly connected proteins would have extremely variable effects on splicing efficiency, but these effects will be higher if poorly-connected proteins interact with highly-connected, functionally important proteins. Thus, asymmetrical interactions underlying the nested pattern may help understanding the effects of auxiliary proteins, transiently associated to the spliceosome. Auxiliary proteins usually interact with a core element and disrupting these interactions interferes with splicing regulation. In yeast, the protein Cwc24p interacts with only a few proteins, but its absence blocks splicing on RNA transcripts that do not have consensus branchpoint sequences^30^. Therefore analysing the structure of spliceosome networks can prove important to understand the dynamics of splicing regulation driven by peripheral proteins. It is possible that other protein-protein networks also have a nested organization, and given the relationship between nestedness and robustness, we consider this question warrants future research. In fact, the asymmetries underlying nestedness may help to explain why some poorly-connected proteins show high lethality and the importance of functional centrality in protein-protein interactions^31^.

When we considered only interactions with higher reliability (cutoff = 0.5), we found smaller but still significant nestedness degree and higher modularity. Modularity is also associated with network robustness since perturbations are less likely to spread in modular networks^5^,^32^. In the spliceosome network modules seem to be related to functional groups and may represent different steps in spliceosome assembly. For example, components of the Prp19-complex (NTC) are assigned to one module, which also contains proteins recruited at the second step of splicing catalysis, such as Prp18p, Prp22p and Slu7p. This might be due to the fact that both groups of proteins are associated to the spliceosome at the same stage of maturation, after B complex formation^33^. NTC proteins associate to spliceosome soon in B complex and play an important role on stabilization of snRNAs, facilitating catalytic activation of the spliceosome^34^,^35^. At the same time, helicases Prp16p and Prp18p are recruited for the completion of the second step of splicing^36^. These are proteins recruited and concentrated on spliceosome complexes B and C, especially important for catalysis. Tri-snRNP components and U5 snRNP proteins (Prp8p and Brr2p) also form a module that is probably temporally associated, since these proteins associate with the complex at the same time^33^,^37^. Interestingly, this module is separated from the second step factors, clustered with the NTC subcomplex, despite the similar timing of association.

The other two modules we found are related to U2 snRNP proteins and U1 snRNP. Importantly, U2 snRNP is remodeled during spliceosome activation^38^ and some U2 components remain associated to the U2-U6 pair at the catalytic core of the complex, as already observed by mass spectrometry analysis^39^,^40^. Almost all proteins in the U2 snRNP module are also associated to 17S/U2 particle. The module containing the components of U1 snRNP includes proteins recruited early in A complex^33^ and probably reflects a temporal association between these proteins in the spliceosome.

Our results using food-web models support the notion that affinity and specificity are important in the assembly of the spliceosome network. The Probabilistic Niche Model, independent of the level of interaction reliability considered, can reproduce the network structural patterns. In fact, the performance of the PNM in reproducing spliceosome protein-protein interactions (>80% of interactions correctly predicted) is above the average of the success obtained in reproducing interactions among species in food webs (75 ± 13% of species interactions correctly predicted)^23^. Even though we found model goodness of fit increased as we increased model complexity, the one-dimensional model performed well in reproducing network structure, which suggests it may include fundamental network assembly rules.

The simplicity of the one-dimensional model allows the examination of the distribution of model parameters, informing how network structural properties could emerge from the variation in protein traits determining affinity and specificity. For the spliceosome network with interaction reliability cutoff = 0.15, *n*-values are uniformly distributed, suggesting that proteins are spread along the trait axis. The abundance of intermediate *c*-values implies that most proteins will interact with the same subset of proteins, generating redundancy, one of the main components of nestedness^41^. Nevertheless, there is a wide variation in the interaction range. Most proteins have a narrow interaction range and only a few have wide interaction ranges, generating asymmetries, another key component of nested networks^42^.

Insights on how the modular structure can emerge are also encoded in the distribution of PNM parameters. When we considered only interactions with higher reliability (cutoff = 0.5), the distribution of *n*-values was characterized by peaks, likely related to the modules we see in the network. *r*-values are generally small, meaning most proteins have limited interaction ranges. The clustered distribution of proteins around peaks in the trait axis and narrow interaction ranges end up reducing the overlap in interaction patterns and increasing modularity.

The fact that the bulk of the interactions can be reproduced by the model using a single trait axis is noteworthy. In ecological networks, only one or a few trait dimensions are often enough to predict the majority of interactions despite the diversity of functional traits shaping ecological interactions^43^. This occurs because the effects of multiple ecological traits are often correlated and, therefore, a few traits such as body size^44^ characterize much of the organization of food webs. For instance, size effects generate nestedness in some ecological networks, such as that formed by predators and prey in African savannahs where the diet of smaller carnivores is a subset of the diet of hyaenas and lions^45^. In protein-protein interactions, the physical and biochemical characteristics of each protein are often the biological mechanisms underlying affinity and specificity. Although the relationship between protein mass and connectivity was not that marked, our data suggest that the largest proteins (higher molecular weight; MW) have more partners, probably because of greater available surface and interaction possibilities. For instance, Prp8p (279.5 kDa) and Brr2p (246.2 kDa) are among the proteins with more interactions. In a dynamic complex system such as the spliceosome, several transient interactions among proteins might occur during assembly. In fact the interactions of highly connected proteins probably do not occur simultaneously; rather, most of these might occur transiently, but only a few remain, forming defined protein cores^1^,^37^. On the other extreme, proteins with lower molecular weight tend to have fewer partners; for instance Cwc24p (29.7 kDa), Cwc25p (20.4 kDa) and Cwc15p (19.9 kDa) interact with less than 10 proteins in our network. Thus, differences in protein size along with differences in the timing of association of each protein to the complex, which seem to determine module membership, are likely important in network assembly and are potential candidates to represent the dimensions of the PNM.

Food-web models have brought insights into the similarities between ecological and social systems^46^. Here we show that the model able to reproduce empirical food webs can also reproduce protein-protein interactions in a sub-cellular level of organization in a large macromolecular complex. The good performance of the food-web model is surprising due the disparate evolutionary processes molding protein and ecological interactions. The assembly model used here does not allow inferring the evolutionary processes determining specificity and complementarity. However, different evolutionary routes could be leading to the same similar assembly rules. For example, the organization of protein-protein networks is, at least in part, a consequence of natural selection favoring cooperation between proteins in achieving a given task^47^. In contrast, in ecological networks, patterns of interaction emerge, at least in part, as a by-product of natural selection shaping the evolution of local populations of different species^48^ through a myriad of ecological interactions, such as competition, predation or mutual benefit based on reciprocal exploitation (mutualism). Game-theory models for networks predict that the evolutionary consequences of the same rule are markedly distinct if the system is formed by cooperators (as proteins in a protein network) or defectors (as most trophic interactions in a food web)^49^. Future work should explore how complementarity and specificity rules emerge from the disparate evolutionary processes underlying biological networks.

Beyond the insights on network assembly, food-web models allow the reconstruction of biological networks with realistic structure *in silico*, and so such models are useful to examine the functioning of ecological communities^50^ and the implications of their disassembly due to species loss^51^. The good fit of the PNM to a protein-protein network endorses the use of a similar approach, based on network models, to study the dynamics of complex subcellular systems. Proteins related to different modules, for instance, might have different roles in stabilizing or maintaining the whole set of interactions in the network. Probabilistic network models allow reconstructing an ensemble of networks with structural properties that mimic real protein-protein interaction systems while incorporating our uncertainty on interaction incidence. This set of similar networks can be used to test the interdependence of the modules to splicing dynamics or to identify how dysfunctional mutations on genes related to different parts of the complex lead to the fragmentation of the network. Adapting or developing models inspired in food-web models can thus open new avenues for the analysis of the functioning and vulnerability of macromolecular interactions.

## Methods

### Spliceosome protein-protein network

The spliceosome is a multi-megadalton complex responsible for catalysis of splicing in eukaryotic cells. This is an essential process for gene expression regulation, removing intermediate sequences (introns) and ligating exons in a sequential order to generate mature RNA molecules^8^. The spliceosome is a conserved macromolecular machinery, with orthologous proteins identified from yeasts to humans^9^,^52^. More than 100 proteins, along with five snRNAs (U1, U2, U4/U6 and U5), participate in this complex assembly in the yeast *Saccharomyces cerevisiae*. The catalytic activation of the spliceosome, as well as the regulatory properties of its components, derives from the dynamics of interactions among these proteins and the RNAs. Here we used interaction data from the spliceosome protein interaction map of *S. cerevisiae* available in the STRING database (http://string-db.org). Interactions between proteins are inferred from physical and biochemical analysis, generating a confidence value, which depends on how strong are the interactions. Thus a positive protein-protein interaction means that this pair of proteins can be co-precipitated at some point during spliceosome assembly.

To investigate if our results for spliceosome are not restricted to data from a particular database, studied species and the criteria used to establish protein interactions, we also analyzed two additional datasets, one from *S. cerevisiae* and the other from the human spliceosome. These datasets were obtained from Uniprot (http://www.uniprot.org) and included data from 5 different databases (BioGRID, DIP, IntAct, MINT and STRING). The great majority of the proteins and interactions in these additional datasets are core components of the spliceosome catalytic complex and do not include the many transient interactions present in the main dataset. Among these transient interactions are, for example, cap-binding complex proteins, like CBC2. Also, proteins related to RNA metabolism, such as RPL30, NPL3, NAM8 and PML1. Because the datasets include only the core components of the spliceosome, the second *S. cerevisiae* network can be considered a subset of the larger analyzed network. In fact, all proteins present in this second dataset are also present in the main *S. cerevisiae* network.

Interactions between the spliceosome proteins can be represented as an *m* × *m* adjacency matrix **A**, where the rows and columns represent spliceosome proteins, and *a*_*ij*_ = 1 if proteins *i* and *j* interact and *a*_*ij*_ = 0 otherwise. Ideally, *a*_*ij*_ = 1 would imply that the interaction occurs. However, different methods available to study protein-protein interactions provide support for the occurrence of protein-protein interaction. The STRING database provides a score combining the evidence derived from different types of experiments^53^. This reliability score varies from zero (no empirical evidence for the interaction) to one (strong empirical support for interaction). To explore the organization of the empirical network we incorporate this uncertainty in our analyses by using an approach previously used to characterize weighted ecological networks^25^. We assumed two proteins interact if the STRING score for the interaction was higher than a given cutoff. To find the most informative cutoffs we examined how the eigenvalues of **A** vary with changes in the cutoff^54^. This analysis suggested there are two main configurations depending on the cutoff value chosen to build the adjacency matrix (Supplementary Methods). These different configurations are also clear when we analyze the structural properties (see below) of networks built under a series of successive cutoffs (Supplementary Methods). Therefore, to account for these different possible network configurations, we analyzed the spliceosome network with two cutoffs of interaction reliability: a permissive 0.15 and a restrictive 0.5 level of support for interactions.

### Network structure

To characterize network structure we measured two network properties that describe how the interaction patterns of proteins overlap: nestedness and modularity. We are particularly interested in investigating if: (1) nestedness is also observed in a protein-protein network; (2) the notion that modularity characterizes protein-protein networks is valid for the spliceosome network. Nestedness is high (closer to 1) when the interactions of proteins with low connectivity are subsets of the interactions established by more connected proteins. To compute nestedness we modified a metric widely used in the analysis of two-mode networks: NODF^41^. NODF is based on pairwise comparisons between nodes with different number of interactions in each matrix dimension (rows and columns separately). Because the spliceosome network is a one-mode symmetric network, total nestedness is the average degree of pairwise nestedness considering all pairwise comparisons over one matrix dimension.

Modularity increases (towards 1) when the network is comprised of multiple groups of proteins densely connected but with fewer connections to proteins of other groups. We measured modularity using the metric *M* computed with a combination of a fast-greedy and a simulated annealing algorithm in MODULAR^55^. We also registered the proteins assigned to each module to explore the relationship between structure and function in the spliceosome network.

Nestedness and modularity are partially generated by basic structural features of networks such as the number of proteins and the number and heterogeneity of interactions among proteins. To test if the spliceosome network was more nested or more modular than expected by these basic properties, we compared the empirical NODF and *M* values with the values recorded for an ensemble of theoretical networks. We generated such networks using a null model that controlled for the number of proteins and the number and heterogeneity of interactions per protein (adapted from null model 2 in Bascompte *et al.*^13^). This null model assumes the probability of an interaction between proteins *i* and *j* depends on the number of interactions, *k*_*i*_ and *k*_*j*_, they establish with all *m* proteins in the network:


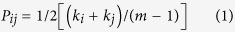


Thus, proteins that are highly connected in the observed network have higher probabilities of being assigned interactions in the theoretical networks generated by this model. Because the number of interactions per protein is variable in the empirical network (see Fig. 1) the null model 2 generate highly heterogeneous networks as generated by other theoretical models, such as the Barabási-Albert preferential attachment model^56^.

To better understand the mechanisms determining protein interaction patterns, we tested the relationship between the number of interactions of each protein (protein connectivity) and its molecular weight. We used linear least squares regression and randomization tests to assess the significance of regressions. Additionally, we tested whether protein connectivity was correlated in the two alternative network configurations (cutoff 0.15 and 0.5), which would mean most information on the interaction patterns of proteins was conserved despite the two different criteria used for building the network.

### Food-web model

We used the probabilistic niche model (PNM)^14^,^23^ to test whether the food-web assembly rules would reproduce the architecture of a network of interacting proteins in the spliceosome. We chose to use a version of the niche model^57^, which is based on simple but general rules and has been the main food-web model used in ecological studies^50^,^51^. The niche model is based on the niche concept, which is a key concept in ecology, and helped unravel the mechanisms underlying network structure^14^,^43^,^57^, investigating the dynamics of ecological communities^50^,^51^ and has been used as the basis for the construction of other models^20^,^58^. The probabilistic version of the niche model is an important improvement over the original version^57^, which treated interactions in a deterministic way. Moreover the probabilistic version allows the computation of model likelihood^23^. The PNM assumes species can be ordered along axes representing niche dimensions and the probability a consumer will use a given resource depends on the position the resource occupies in these axes and the consumer dietary breadth^14^,^23^. Each niche dimension can be viewed as emerging from multiple biological factors (e.g., diet preferences, morphology, behavior) shaping the probability of interaction between species^43^. The probability of an interaction between species *i* and *j* is a continuous function:


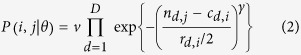


where *n*_*d,j*_ represents the position in the niche dimension *d* for species *j*, *c*_*d,i*_ represents the diet optimum of consumer *i* for dimension *d*, *r*_*d,i*_ is the diet range for species *i* within dimension *d*, γ controls the cutoff rate of the probability function, and *ν* is the maximum probability that *i* consumes any given prey. Thus, species with wider diet ranges have a higher probability of interacting with a wider set of species.

Similarly, proteins might have different specificities to interact with other proteins; some proteins such as Cwc24p, are very specific, interacting with a small set of proteins, whereas others, such as Prp8p, interact with multiple proteins during spliceosome assembly^1^,^26^. Accordingly, the multiple factors that determine protein specificity and affinity can be, in theory, represented as axes. The position each protein is assigned along these axes and the center of the interaction range of its partners determine protein affinities, whereas the amplitude of the interaction range determines the specificity of each protein. We tested the model ability in predicting the protein-protein network organization in scenarios assuming variable levels of complexity: from the simplest scenario, i.e., a single axis (*D* = 1) to more complex scenarios (*D* = 2 and *D* = 3).

Food webs are directed networks, in which who is the predator and who is the prey often matters. In protein networks, we are only interested in whether a given protein-protein interaction occurs or not. Thus, the probability that proteins *i* and *j* interact, *Q*_*ij*_ = *Q*_*ji*_, and is determined by the interaction ranges of both *i* and *j*, so that:


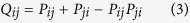


We assumed oligomerization would not be representative of the protein-protein interactions, setting *Q*_*ii*_ = 0. We combined simulated annealing^59^ and Latin hypercube sampling^60^ to find values of *r*_*i*_, *c*_*i*_ and *n*_*i*_ maximizing model likelihood^14^. To assess the model performance in reproducing interactions between splicing proteins we computed the expected fraction of interactions (and absent interactions) predicted correctly:


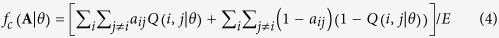


where *E* = *m* × (*m* − 1) is the total number of potential interactions in the network (off-diagonal cells in matrix **A**). Considering only the first term of eq. 4 and dividing by the number of observed interactions we obtain the expected fraction of observed interactions predicted correctly (*f*_*c_obs*_).

We compared the goodness of fit of the PNM to the spliceosome data to that of two simpler network models. The first model is a random Erdős Rényi model, which assumes all interactions are equiprobable. The second model incorporates the observed heterogeneity in the number of links and it is the same we used to test the significance of network structural patterns (see above). We used AIC to compare the goodness of fit of all models while accounting for the differences in the number of model parameters. To test whether models were able to reproduce network structure we built binary networks (100 per model) from the probability matrices generated by each model and computed nestedness and modularity of each network to obtain distributions of nestedness and modularity values for each model. The empirical degrees of nestedness and modularity were considered significantly different from those computed for theoretical networks if falling outside of the 95% confidence intervals of these distributions.

## Additional Information

**How to cite this article**: Pires, M. M. *et al.* The network organization of protein interactions in the spliceosome is reproduced by the simple rules of food-web models. *Sci. Rep.* **5**, 14865; doi: 10.1038/srep14865 (2015).

## Supplementary Material

Supplementary Information

## Figures and Tables

**Table 1:** Performance of the probabilistic niche models (PNM) and null models in reproducing the spliceosome network.

Model	Cutoff = 0.15				Cutoff = 0.50			
	*f*_*c*_	*f*_*c_obs*_	AIC	ΔAIC	*f*_*c*_	*f*_*c_obs*_	AIC	ΔAIC
3D-PNM	0.91	0.88	0.47 × 10^4^	0	0.94	0.76	3.72 × 10^3^	0
2D-PNM	0.88	0.86	0.50 × 10^4^	0.03 × 10^4^	0.92	0.72	4.00 × 10^3^	0.28 × 10^3^
1D-PNM	0.81	0.8	0.70 × 10^4^	0.23 × 10^4^	0.87	0.60	5.43 × 10^3^	1.71 × 10^3^
Null2	0.62	0.59	1.08 × 10^4^	0.61 × 10^4^	0.76	0.26	7.83 × 10^3^	4.11 × 10^3^
Null	0.51	0.48	1.45 × 10^4^	0.98 × 10^4^	0.73	0.16	9.50 × 10^3^	5.78 × 10^3^

Values for the networks built using cutoffs 0.15 and 0.5. Models are ranked by goodness of fit (AIC) and fraction of interactions correctly predicted (*f*_*c*_ and *f*_*c_obs*_).
