# Supplementary material for: The network organization of protein interactions in the spliceosome is reproduced by the simple rules of food-web models
Source: Sci Rep. 2015 Oct 7;5:14865. doi: 10.1038/srep14865 (PMC4595644; doi:10.1038/srep14865)
Supplement: Supplementary Information [file srep14865-s1.pdf]

SUPPLEMENTARY INFORMATION

The network organization of protein interactions in the spliceosome is reproduced by the simple rules of food-web models

Mathias M. Pires, Maurício Cantor, Paulo R. Guimarães Jr., Marcus A. M. de Aguiar, Sérgio F. dos Reis & Patricia P. Coltri

Supplementary Methods

Eigenvalue variation and the reliability of protein-protein interactions

In some networks, such as protein-protein networks, different kinds of experiments provide different levels of evidence supporting the occurrence of a given interaction. As a consequence, a fundamental question for our analysis is how the description of network organization changes with the minimal level of reliability we assume is enough to accept an interaction is occurring (interaction reliability). Here we used the distribution of eigenvalues to explore the sensibility of network description to the degree of reliability.

Any network formed by N nodes can be depicted by an adjacency matrix **A** characterized by N eigenvalues ($\lambda_1 \geq \lambda_2 \geq \lambda_3 \geq ... \geq \lambda_N$). The distribution of eigenvalues (the spectra) of the adjacency matrix **A** provides detailed information on the structure of complex networks[1]. Therefore, the distribution of eigenvalues can be used to detect how network structure changes assuming different cut-off values for interaction reliability[2]. We computed the eigenvalue variation between two consecutive cutoff values for interaction reliability (the STRING score), x_a and x_b, as

$\Delta\lambda = \sum_{i=1}^{N}\left(\lambda_i^a - \lambda_i^b\right)^2 \Big/ N$. If eigenvalue variation is zero, there is no change in network description between consecutive cutoff values, whereas higher values of eigenvalue variation implies in major shifts in network description.

For the spliceosome network there is a clear threshold at cutoff = 0.15 in which network description changes abruptly (Supplementary Fig. S1). After this cutoff, increasingly higher cutoff values for minimal interaction reliability do not markedly affect network description. Thus, after removing interactions with very weak support (cutoff value < 0.15), the network description is robust and we used cutoff value = 0.15 to describe the spliceosome network. We also analyzed the network using the cutoff value = 0.5 representing another robust configuration after a second, less abrupt, threshold around cutoff value = 0.4 (Supplementary Fig. S1). The 0.5 cutoff value also represents a more conservative way of building the network, where only interactions with high reliability are considered.

Table S1. Performance of the probabilistic niche models (PNM) and null models in reproducing the two additional spliceosome networks analyzed. Models are ranked by goodness of fit (AIC) and fraction of interactions correctly predicted (f_c and f_{c_obs}).

	Yeast				Humans			
Model	f_c	f_{c_obs}	AIC	ΔAIC	f_c	f_{c_obs}	AIC	ΔAIC
3D-PNM	0.99	0.99	666.11	441.65	0.99	0.99	1152.3	662.11
2D-PNM	0.99	0.99	444.21	219.75	0.99	0.99	769.00	278.81
1D-PNM	0.99	0.99	224.46	0	0.99	0.99	490.19	0
Null2	0.87	0.13	613.78	398.3	0.96	0.05	1245.5	755.31
Null	0.86	0.07	700.06	475.6	0.96	0.04	1333.4	843.21

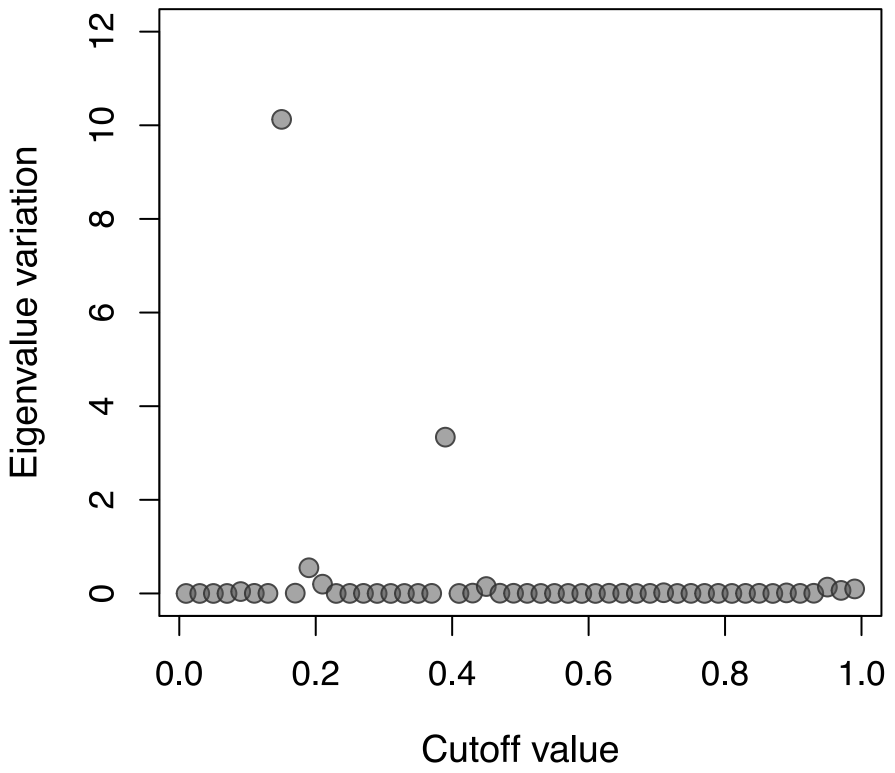

Figure S1. Change in network configuration with cutoff values measured by variation in eigenvalues. We compute the change in the distribution of eigenvalues between consecutive cutoff values for interaction reliability based on STRING Score (see main text). The higher the eigenvalue variation, the higher the change in network structure with the change in interaction reliability.

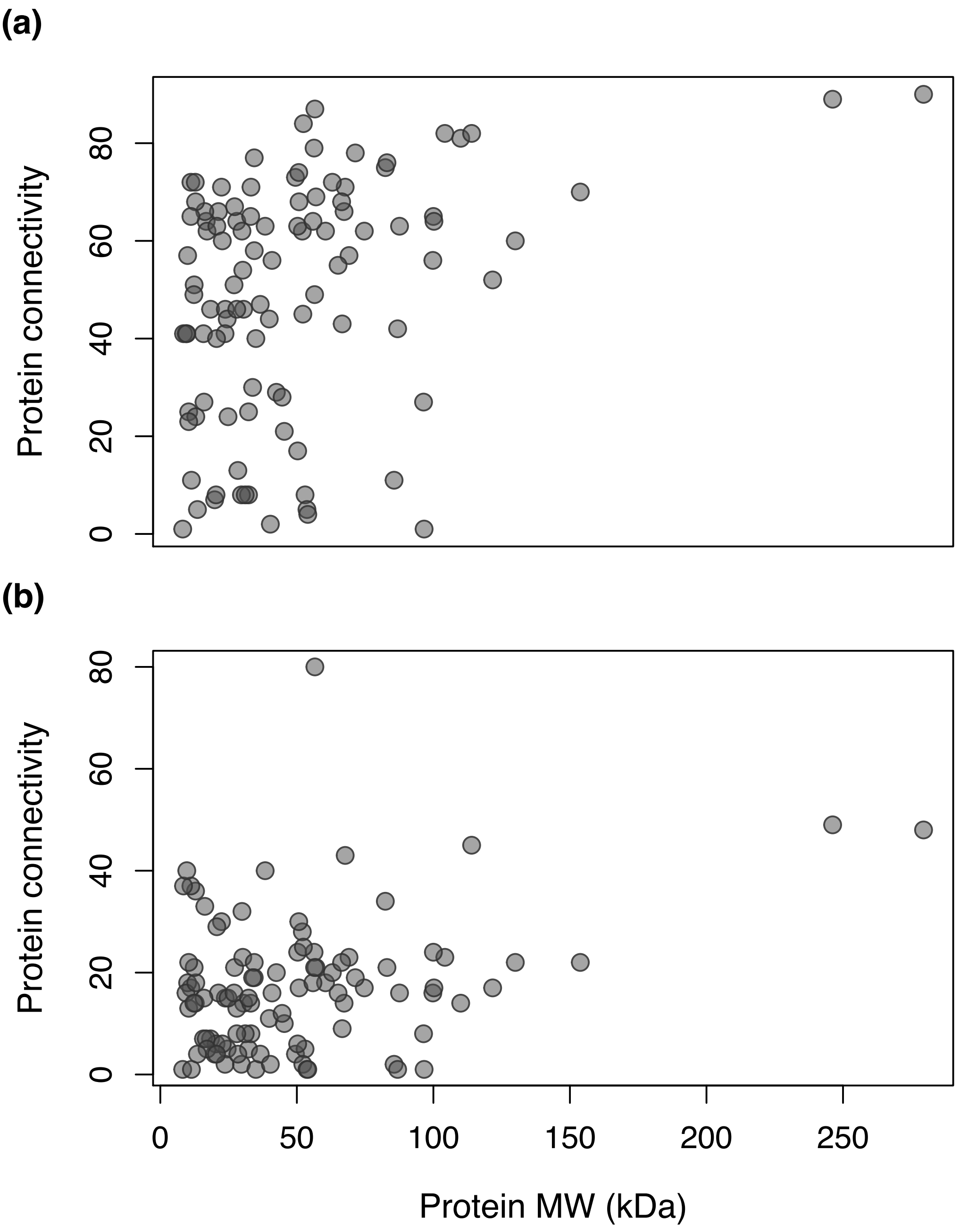

Figure S2. Relationship between the number of interactions of each protein (protein connectivity) and its molecular weight, using cutoff = 0.15 (**a**) and cutoff = 0.5 (**b**) of reliability to define interactions in the network.

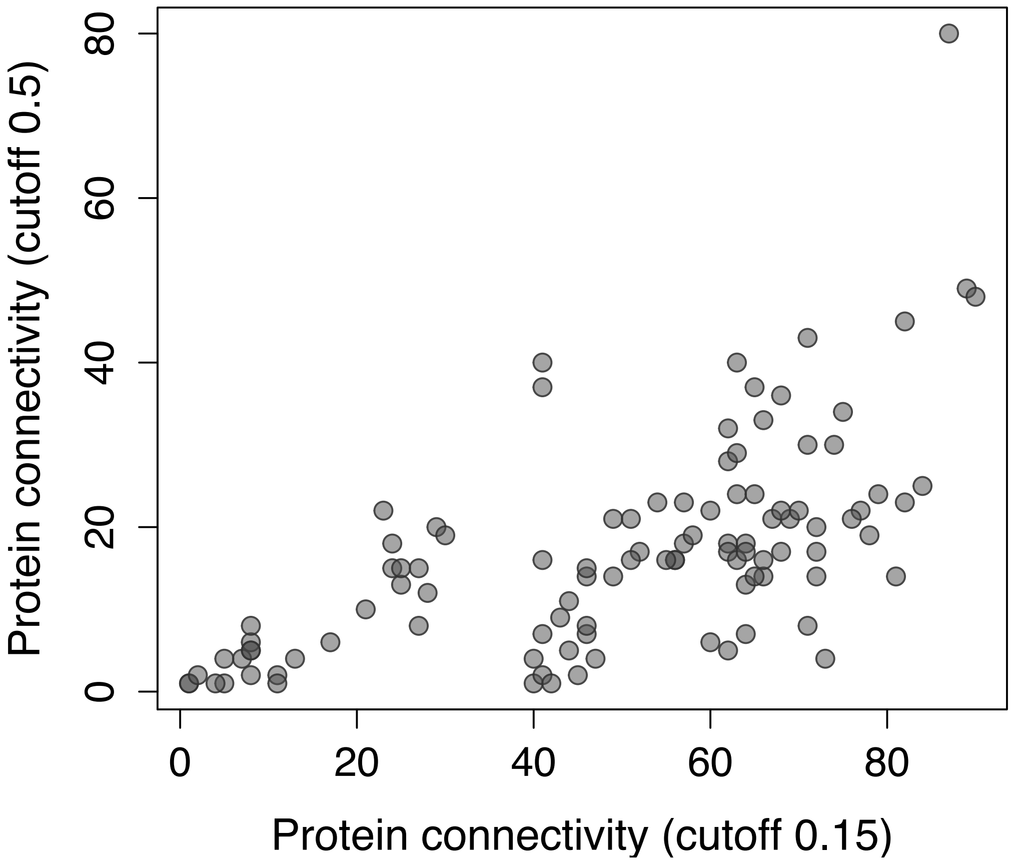

Figure S3. Relationship between protein connectivity in the network built under cutoff 0.50 and cutoff 0.15.

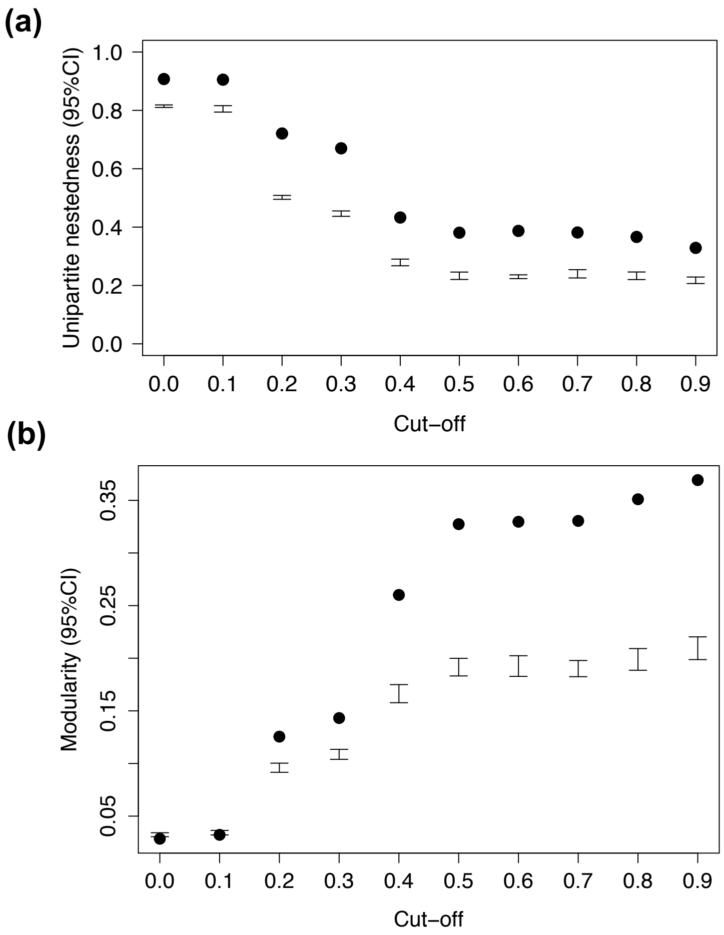

Figure S4. Variation in nestedness (**a**) and modularity (**b**) for networks built according to varying cutoff values. Bars denote the 95% confidence interval for null networks built under the assumption that the probability of interactions is proportional to the number of interactions of potentially interacting proteins.

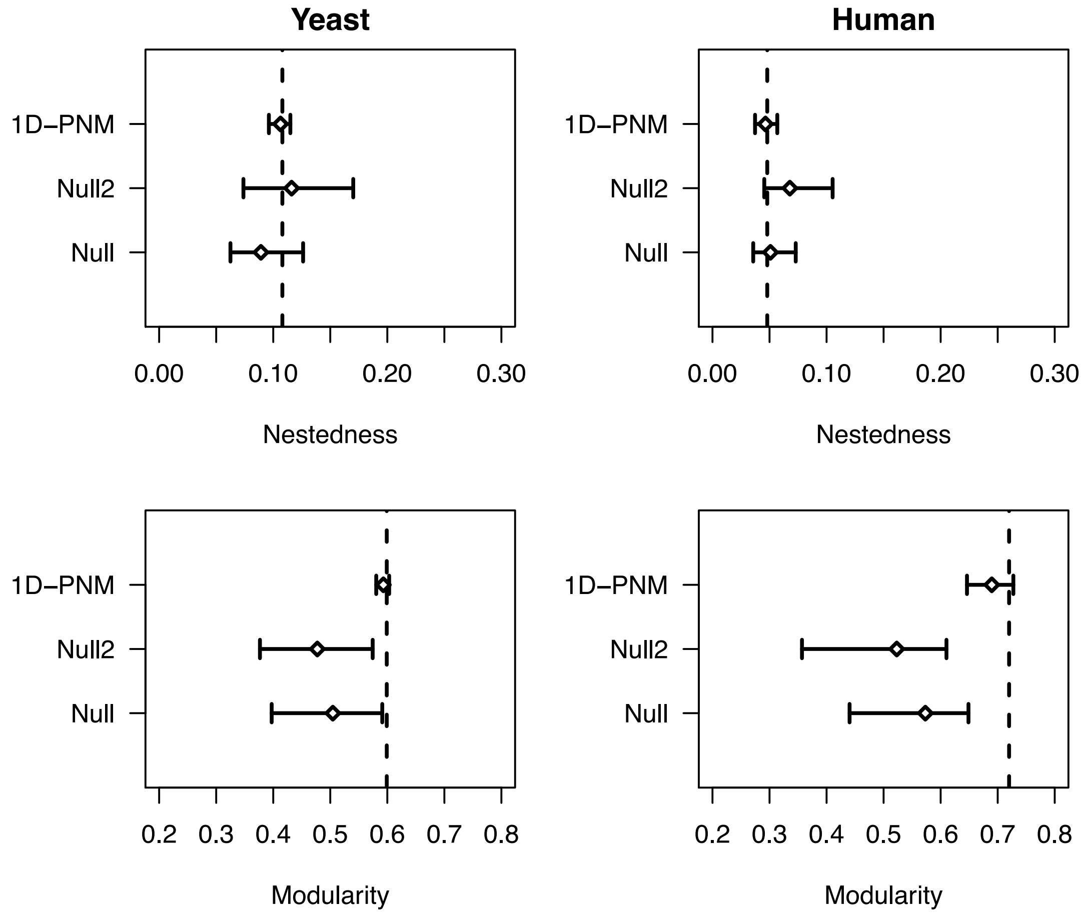

Figure S5. Nestedness and modularity of the two additional networks analyzed. Theoretical networks built according to two null models and the one-dimensional probabilistic niche model, 1D-PNM. Whiskers = 95% CI. The dashed lines represent estimates for the observed networks.

References

1. Aguiar, M. A. M. & Bar-Yam, Y. Spectral analysis and the dynamic response of complex networks. *Phys. Rev. E* **71,** e016106 (2005).
2. Perez, S. I., Aguiar, M. A. M., Guimarães, P. R. & Reis, S. F. Searching for modular structure in complex phenotypes: Inferences from network theory. *Evol. Biol*. **36,** 416-422 (2009).
